# In Situ Constructing Robust Interface by Deep Eutectic Polymeric Electrolyte Enables High Performance Lithium Metal Batteries with High‐Loading Cathode

**DOI:** 10.1002/advs.202411421

**Published:** 2024-10-28

**Authors:** Zixuan Fang, Ming Zhang, Zhihao Zhang, Jitao Li, Haofeng Peng, Jintian Wu, Haiping Zhou, Ziqiang Xu, Mengqiang Wu

**Affiliations:** ^1^ School of Materials and Energy University of Electronic Science and Technology of China Chengdu Sichuan 611731 China; ^2^ School of Precision Instruments and OptoElectronics Engineering Tianjin University Tianjin 300072 China; ^3^ School of Chemical Engineering Sichuan University of Science & Engineering Zigong 643000 China; ^4^ Yangtze Delta Region Institute (HuZhou) University of Electronic Science and Technology of China Huzhou Zhejiang 313001 China

**Keywords:** anchoring effect, deep eutectic electrolytes, high‐loading cathode, lithium metal batteries, solid state batteries

## Abstract

The low Li^+^ transport and poor interface have consistently been two major impediments to practical applications of Polyacrylonitrile (PAN)‐based composite solid‐state electrolytes (PCPE). In this work, a polymerizable deep eutectic electrolyte is meticulously designed with high fluidity which consists of Poly (Ethylene Glycol) Diacrylate (PEGDA), Fluoroethylene Carbonate (FEC), Succinonitrile (SN) and dual salts (LiTFSI/LiDFOB) to promote Li^+^ transport and ameliorate the interface of PCPE. Inclusion of PEGDA monomers and FEC alters the crystallinity of SN, enhancing the wettability of thick electrode, and formation of polymeric 3D network from polymerization of PEGDA can anchor SN and suppress the side reactions between SN and lithium metal. Consequently, the modified PCPE exhibit an enhanced conductivity of 4.47 × 10^−4^ S cm^−1^ with Li‐ion transference number of 0.60, and show an excellent lithium stability. LiCoO_2_(LCO)/SP‐PCPE/Li batteries with higher loading (3–4.4 V, 6 mg cm^−2^) can work for over 300 cycles at 0.5 C. Even with an ultra‐high loading of 16 mg cm^−2^, LCO/SP‐PCPE/Li batteries achieve an excellent cycling performance. This work provides new insights into how to construct a robust interface for solid‐state batteries with high‐loading cathode.

## Graphical abstract

A polymerizable deep eutectic electrolyte with high fluidity is elaborately designed to promote Li^+^ transport and ameliorate the interface of (PAN)‐based composite solid‐state electrolytes. The interfacial eutectic polymerization strategy effectively enhances the compatibility of the SP‐PCPE with high‐voltage and high‐loading cathodes, and Lithium metal, giving rise to the excellent performance of solid‐state batteries.

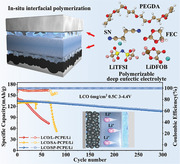

## 1 Introduction

The rapid growth in market of electric vehicles, 3C electronics and outdoor portable energy storage power supplies has stimulated the huge demand for Lithium‐ion batteries (LIBs). However, with the urgent pursuit of energy density higher than 500 Wh kg^−1^ and rising concerning on safety issues, LIBs have encountered a series of challenges in their continuous development. In particular, the flammable liquid electrolyte used in LIBs performs poorly in terms of electrochemical stability, which not only suffers from a potential safety risk, but also limits its compatibility with high‐voltage cathodes and lithium‐metal anodes.^[^ ^1^ ^]^ In the past decades, solid‐state electrolytes, with their excellent electrochemical stability and wide electrochemical window, have become the key to solving the aforementioned problems, and solid‐state batteries are widely regarded as candidates for the next generation of battery technology.^[^ ^2^ ^]^ Therefore, an in‐depth research of solid‐state electrolytes has become a vital pathway for achieving technological breakthroughs in the field of energy storage.

Composite solid electrolytes (CPE) made of organic and inorganic materials combine the high flexibility of organic electrolytes with the high ionic conductivity and excellent mechanical properties of inorganic electrolytes, showing great potential for commercialization.^[^ ^3^ ^]^ Among them, PAN‐based CPE (PCPE) has excellent oxidation resistance, which are able to match the common high‐voltage cathode because of the wide electrochemical stability window.^[^ ^4^ ^]^ The high mechanical strength of PAN can effectively inhibit the growth of lithium dendrite during the deposition/stripping process, which makes PCPE the ideal alternative electrolytes for solid‐state batteries with high‐voltage cathode.^[^ ^5^ ^]^ Nevertheless, the high crystallinity of PAN greatly restricts the movement of polymer chains within the matrix, which limits the ion transport in PCPE^[^ ^5^, ^6^ ^]^ and deteriorates the electrolyte/electrode interfacial impedance.^[^ ^7^ ^]^ The limited ion transport performance and poor interfacial contact make it difficult for solid‐state batteries assembled with PCPE to cycle stably.

Interfacial modification strategies have been employed to reduce interfacial impedance and ensures high ion transport.^[^ ^8^ ^]^ Intensive works have been devoted to wetting the interface between CPE and electrode by utilizing liquid electrolyte (LE). But the consumption of liquid electrolytes in long‐term charge‐discharge cycles, along with the instability of the solid‐liquid interface, leads to the continuous degradation of battery capacity.^[^ ^9^ ^]^ To alleviate the serious consumption issues for PCPE, various methods were proposed to establish a stable in‐situ solidified interlayer, such as cross‐linked plastic crystal electrolytes, eutectic electrolytes, etc.^[^ ^5^, ^10^ ^]^ Due to the nature of plastic crystal, succinonitrile (SN) can be utilized to synthesize different kinds of eutectic electrolytes. Importantly, the eutectic electrolytes, composed of metal salts and SN with abundant polar‐CN groups enabling SN to play an excellent plasticizing role on PAN with garnet type (Li_6.4_La_3_Zr_1.4_Ta_0.6_O_12_) inorganic fillers according to our previous work,^[^ ^11^ ^]^ can tolerate degradation during battery cycling and construct a stable interface. Despite the potential advantages of SN based eutectic electrolyte in PAN‐based systems, its high viscosity and crystallinity at room temperatures still constrain its wetting effect on thick cathode (high‐loading cathode), affecting the cycling performance of the battery,^[^ ^10^, ^12^ ^]^ Additionally, the high reactivity of SN with lithium metal increases the risk of side reactions which are difficult to completely avoid in practical operations.^[^ ^13^ ^]^ In situ thermal polymerization,^[^ ^13c^ ^]^ as an alternative to the room‐temperature curing of SN, is promising to address the aforementioned issues by improving wettability of thick cathode and achieving controllable solidification.

Herein, we propose to construct a robust 3D polymeric interface using a polymerizable deep eutectic electrolyte with high fluidity which is composed of PEGDA, SN, LiTFSI/LiDFOB, and FEC. We incorporate PEGDA along with 10% vol FEC into the mixture of SN and LiTFSI/LiDFOB to create a precursor solution characterized by high fluidity and wettability. The incorporation of PEGDA and FEC is to alter the crystallinity of SN, leading to the formation of a deep eutectic precursor. After free radical polymerization, this precursor is transformed into robust 3D polymeric interface with high ionic conductivity (3.1 × 10^−4^ S cm^−1^), designated as SP (PEGDA+10% vol FEC+SN+LiTFSI/LiDFOB), which is intended to improve the unfavorable interface of PCPE. This enhancement not only bolsters the stability of PCPE against lithium metal, but also enhances compatibility with high‐voltage and high‐loading cathodes, thereby facilitating the practical application of PAN‐based CPEs in solid‐state batteries. Comprehensive characterizations, in conjunction with theoretical calculations, further confirm the existence of a stronger interaction between PEGDA, FEC and SN, which effectively promotes the dissociation of lithium salts. Within the SP, SN acts effectively as a plasticizer, endowing the eutectic polymerization‐modified SP‐PCPE with an elevated ionic conductivity of 4.47 × 10^−4^ S cm^−1^ and a high lithium ion transference number of 0.60. Molecular Dynamics (MD) simulations and Density Functional Theory (DFT) calculations indicate that PEGDA exerts an effective anchoring effect on SN, reducing the diffusion coefficient of SN at the SP‐PCPE interface. This anchoring effect effectively restricts the diffusion of SN towards the surface of lithium metal. Additionally, the lower LUMO energy level of PEGDA further mitigates side reactions between SN and lithium metal. Owing to aforementioned beneficial effects, the Li/SP‐PCPE/Li symmetric cell can stably cycle for up to 3600 h at 0.1 mA cm^−2^, and possesses a critical current density of 2.2 mA cm^−2^. The interfacial eutectic polymerization strategy enhances the compatibility of the SP‐PCPE with high‐voltage, high‐loading cathodes, enabling the LCO/SP‐PCPE/Li battery to stably cycle for 300 cycles at 0.5 C (3.0–4.4 V, 6 mg cm^−2^). Remarkably, even under an ultra‐high loading of 16 mg cm^−2^, the LCO/SP‐PCPE/Li batteries achieve an excellent cycling performance during the voltage range of 3.0–4.4 V. This work paves the way for the practicality of PCPE, which is suitable for large‐scale applications.

## 2 Results and Discussion

### 2.1 Designing Strategy of the Deep Eutectic Polymeric Interface

**Figure 1:**
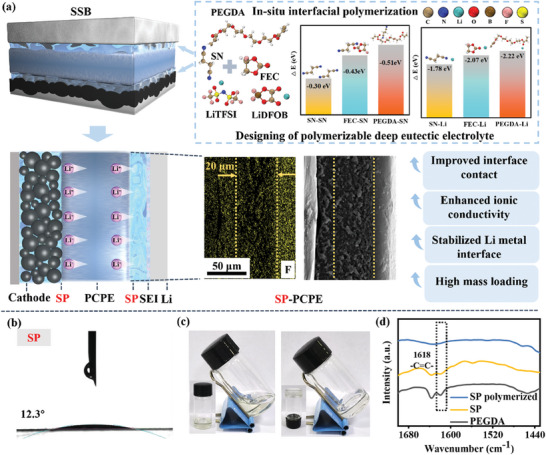
a) Schematic illustration of the eutectic polymerization of SP improving the interface of PAN‐based CPE and the intermolecular and ionic interaction energies; b) Contact angle test between SP and LCO; c) Optical photographs of SP before and after polymerization; d) FTIR spectra of PEGDA and SP before and after polymerization.

**Figure** **1**a illustrates the schematic of in‐situ construction of a robust interface via a polymerizable deep eutectic electrolyte to enhance the PCPE. Deep eutectic electrolytes (DEEs) are a subclass of deep eutectic solvents (DESs). Typically, DESs are prepared from the eutectic mixtures of Lewis acids and bases. The deep eutectic phenomenon occurs when the intermolecular interactions between different components are stronger than the intermolecular interactions within each component. Lithium‐based DEEs, such as those derived from lithium salts (e.g., LiTFSI, LiDFOB), involve the dissociation of Li^+^ ions from the lithium salts through Li···O interactions.^[^ ^14^ ^]^ These intermolecular interactions, sometimes accompanied by hydrogen bonding, lead to the deep eutectic phenomenon. Li···N interactions, which have a similar mechanism to Li···O interactions, can also induce a deep eutectic effect between SN, FEC, PEGDA and LiTFSI/LiDFOB. In our system, the ─CN group of SN and the ether oxygen of FEC act as Lewis bases, while the Li^+^ ions act as Lewis acids. The dissociation of LiTFSI and LiDFOB to form DEE is facilitated by the N atom of SN and the ether oxygen of FEC. The in‐situ polymerization process utilizes highly fluid precursors composed of PEGDA, SN, dual salts (LiTFSI/LiDFOB), and FEC, which can effectively infiltrate the thick cathode. Furthermore, the calculated interactions between SN and PEGDA (SN‐PEGDA, −0.51 eV), as well as SN‐FEC (−0.43 eV) within the solvent polymer (SP), are significantly stronger than those between SN‐SN molecules themselves (−0.3 eV). This finding indicates that the introduction of PEGDA and FEC can effectively disrupt the interactions among SN, resulting in altering the characteristic room‐temperature curing of SN. In our experiments, the addition of 10 vol% FEC alters the characteristic of SN solidifying at room temperature, and the SN system no longer solidifies at ambient conditions (Figure S1a, Supporting Information). The adsorption energies of SN, FEC, and PEGDA with lithium ions are calculated to be −1.78, −2.07, and −2.22 eV, respectively, which confirm that FEC and PEGDA are more conducive to the dissociation of lithium salts. Therefore, the incorporation of PEGDA and FEC also enhances the transport of Li^+^. Furthermore, due to its stronger coordinating effect with lithium ions, FEC can participate in the solvation structure, and the use of FEC as a film‐forming additive has been extensively reported in numerous literature.^[^ ^15^ ^]^ The scanning electron microscopy (SEM) images in Figure 1a demonstrate that the precursors can locally penetrate the interface of the PCPE. Concurrently, SN exerts a plasticizing effect on the PAN molecular chains, which reduces the crystallinity of the matrix, achieves an intimate interfacial contact, and enhances the overall ionic conductivity.^[^ ^11a^ ^]^ Althougth residual DMF is present in PCPE (Figure S2a, Supporting Information), the LLZTO in PCPE system has a strong adsorption energy for DMF (Figure S2b, Supporting Information) according to the calculation of adsorption energies of LLZTO with SN and DMF. This finding indicates that free DMF can be effectively confined within PCPE matrix by LLZTO fillers, and the effect of free DMF on interficial modification is negligible.

Figure 1b displays the excellent wetting behavior of the SP precursor on high loading LCO (6 mg cm^−2^), and it shows a much smaller contact angle of 12.3° in comparison to that of 19.7° for the ordinary SN‐based eutectic additives (SA, SN+5 vol.% FEC+LiTFSi/LiDFOB) and that of 14.9° for conventional liquid electrolyte on LCO (Figure S1b, Supporting Information).^[^ ^10^, ^16^ ^]^ The polymerized PEGDA within the precursors effectively restricts the diffusion of SN at the interface, diminishing the chemical reactivity of SN with lithium metal and suppressing side reactions between SN and lithium metal, thereby improving the stability of the SP‐PCPE towards lithium metal. SPs with varying PEGDA contents (1.5, 3, 6, 12, 24 wt.%) were also analyzed (Figure S3, Supporting Information) in detail, and only the SP containing 24 wt.% PEGDA is able to transform from liquid state solid state after heating at 60 °C for 30 min (Figure 1c), whereas the SA system (without PEGDA and a substantial amount of FEC) solidified at room temperature (Figure S3, Supporting Information). This result indicates that the optimal concentration of PEGDA required for SP polymerization is 24 wt.%. This specific SP is used as the subsequent eutectic polymeric liquid to improve the interface of PAN‐based CPEs, ultimately forming an SP‐PCPE electrolyte. To further investigate the chemical structures of the SP, Fourier Transform Infrared Spectroscopy (FTIR) was employed to characterize the monomer, SP precursor, and post‐polymerized SP, respectively. As shown in Figure 1d, the characteristic peaks of the vinyl groups (C═C) at 1618 cm^−1^ for PEGDA and pre‐polymerized SP are undetectable in the spectrum of the post‐polymerized SP, which confirms the complete free‐radical polymerization.^[^ ^17^ ^]^

### 2.2 Beneficial Mechanism Resulting from Deep Eutectic Polymeric Interface

**Figure 2:**
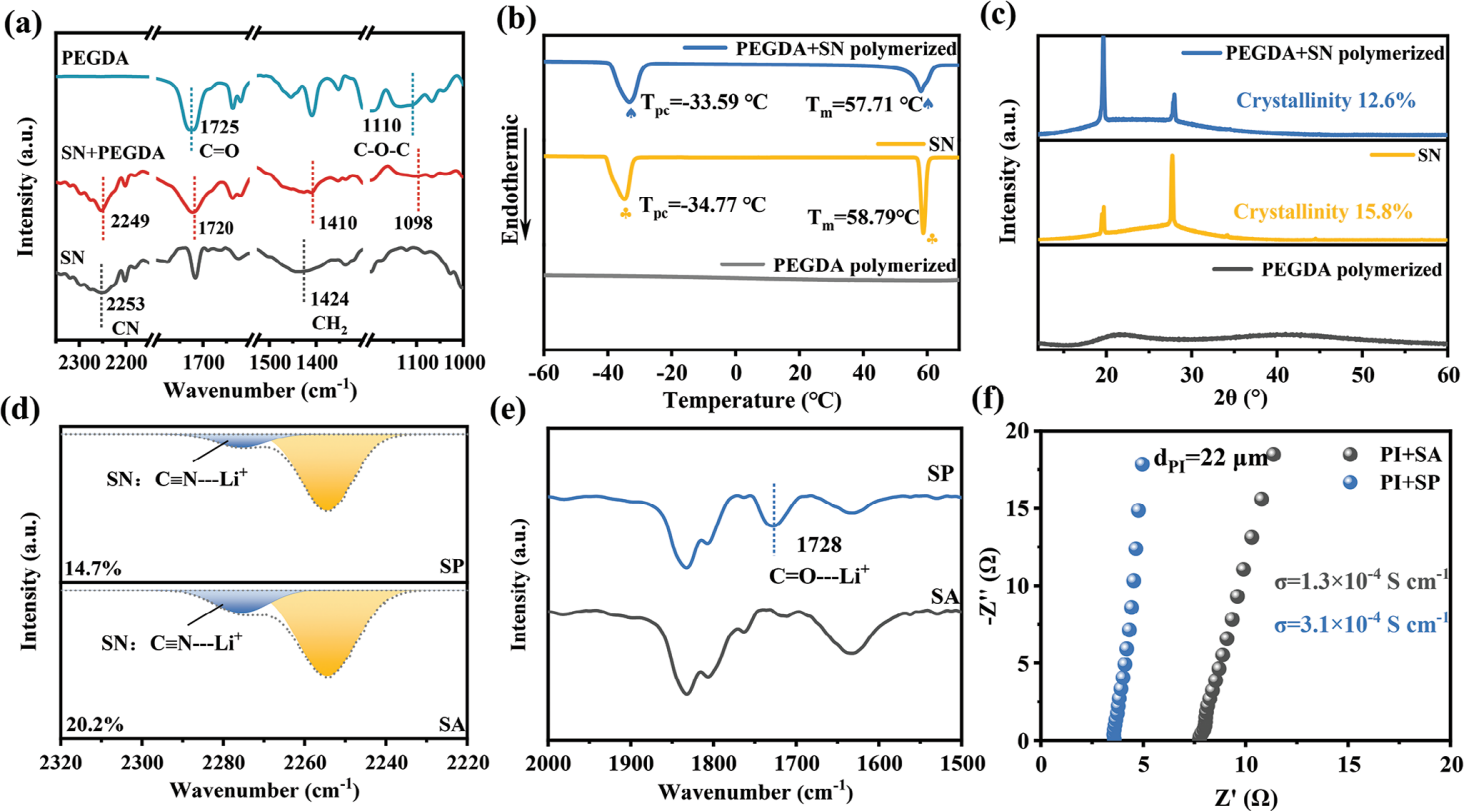
a) FTIR spectra of SN, PEGDA, and their mixture. Spectra of SN, polymerized PEGDA, and post‐polymerized SN+PEGDA: b) DSC profiles; c) XRD patterns. Relevant test analyses for SA and SP: d) FTIR spectra; e) Raman spectra; (f) FTIR spectra of other wavenumber regions; g) EIS impedance tests.

FTIR and Raman spectrum were also utilized to analyze the interactions between SN and PEGDA within the SP. As depicted in **Figure** **2**a, the peaks at 1725 cm^−1^ (C═O) and 1110 cm^−1^ (C─O─C) originated from pristine PEGDA shift to 1720 cm^−1^ and 1098 cm^−1^ in the PEGDA/SN sample.^[^ ^18^ ^]^ At the same time, the peaks of SN at 2253 cm^−1^ (─CN) and 1424 cm^−1^ (─CH_2_) on the spectra of SN experience a redshift to 1720 and 1098 cm^−1^ in the PEGDA/SN sample.^[^ ^19^ ^]^ These results indicate that there is indeed a strong interaction between SN and PEGDA, primarily stemming from the strong polar functional groups such as CN and C═O. Differential scanning calorimetry (DSC) was further conducted to confirm the interaction between SN and PEGDA. No obvious peak can be observed in DSC curve of PEGDA, indicating that polymerized PEGDA exhibits a fully amorphous state.^[^ ^20^ ^]^ SN shows a melting temperature (T_m_) of 58.79 °C and plastic crystalline transition temperature (T_pc_) of −34.77 °C. The addition of PEGDA results in a gradual shift in T_m_ and T_pc_, which reaches 57.71 °C and −33.59 °C in PEGDA/SN, respectively, confirming notable interaction between PEGDA and SN.^[^ ^21^ ^]^ The results of XRD patterns in Figure 2c and Figure S2c,d (Supporting Information) also indicate that the introduction of PEGDA reduces the crystallinity from 15.8% for pure SN to 12.6% for PEGDA/SN sample, which would form more amorphous regions and facilitating Li^+^ migration in electrolyte.^[^ ^19^, ^22^ ^]^

The interaction between PEGDA and SN is expected to facilitate the fast transport of Li^+^. FTIR spectroscopy was adopted to explore the transport of Li^+^ within ordinary SN‐based eutectic additives (SA) and SP. As shown in Figure 2d, the peak at 2253 cm^−1^ corresponds to the ‐CN in SN, and the peak near 2280 cm^−1^ corresponds to Li^+^ coordinated with ‐CN groups. The relative proportion of CN‐Li^+^ in SA is 20.2%, which is decreased to 14.7% after the introduction of PEGDA. This finding suggests that the introduction of PEGDA can disrupt the coordination between SN and Li^+^, leading to the formation of more free Li^+^ and promotion of the transport of Li^+[^ ^23^ ^]^ Furthermore, a new peak near 1728 cm^−1^ in SP appears after the introduction of PEGDA, as shown in Figure 2e, which corresponds to the coordination between Li^+^ and C═O in PEGDA.^[^ ^24^ ^]^ As for the Raman spectrum, the characteristic peaks at 2275 cm^−1^ corresponding to CN‐Li^+^ in both SA and SP (Figure S4, Supporting Information) show that the peak intensity and area of CN‐Li^+^ in SP are stronger than that in SA, indicating that the introduction of PEGDA can weaken the coordination between SN and Li^+^. The EIS impedance in Figure 2f intuitively characterizes the ionic transport capabilities of SA and SP. The results show that the eutectic electrolyte SP, consisting of PEGDA and SA, possesses a higher ionic conductivity of 3.1 × 10^−4^ S cm^−1^, as compared to that of SN (1.3 × 10^−4^ S cm^−1^). PEGDA also has a competitive interaction with Li^+^ and weakens the coordination of Li^+^ with SN, promoting the dissociation of lithium salts and alleviating the side reaction caused by SN, which be discussed later in depth.

**Figure 3:**
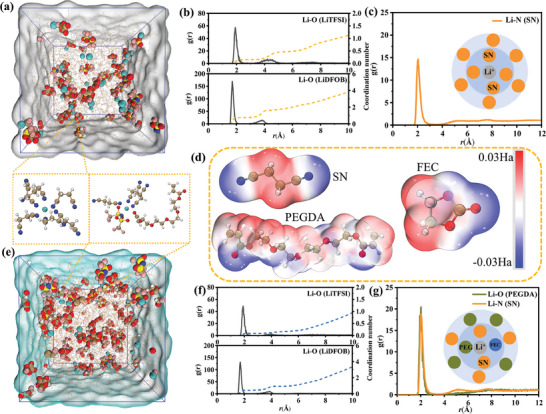
a) Molecular dynamics simulation snapshot of SA; b) RDF of LiTFSI and LiDFOB within SA; c) RDF of SN and Li^+^ in SA; d) ESP of SN, PEGDA, and FEC; e) Molecular dynamics simulation snapshot of SP; f) RDF of LiTFSI and LiDFOB within SP; g) RDF of SN/PEGDA and Li^+^ in SP.

Molecular dynamics simulations were employed to further elucidate the mechanism of Li^+^ transport accelerated by the interaction between PEGDA and SN. **Figure** **3**a–c,e–g presents molecular dynamics simulations for the SN‐based eutectic additives (SA) and the deep eutectic solvent polymer (SP), along with radial distribution functions (RDFs) for LiTFSI and LiDFOB, respectively. As depicted in Figure 3b,f, the coordination numbers for Li‐O(TFSI) and Li‐O(DFOB) in SP are calculated to be 0.10 and 0.48, respectively, which are significantly lower than those for Li‐O(TFSI) (0.16) and Li‐O(DFOB) (0.77) in SA. This finding indicates that the incorporation of PEGDA results in a decrease of the coordination numbers of lithium salts, potentially facilitating their dissociation and increasing the concentration of free Li^+^.^[^ ^12^ ^]^ The solvation structures of Li^+^ with SN and PEGDA were investigated using RDF analysis. Figure 3c illustrates the solvation structure of SN and Li^+^ in the SA, with a distinct peak at 2.05 Å and two broad peaks at 5.05 and 7.65 Å for Li‐NSN, indicating a significant distribution of SN in both the inner and outer solvation shells of Li^+^.^[^ ^25^ ^]^ Figure 3g displays the solvation structure of SN, PEGDA and Li^+^ in SP, with peaks for Li‐N(SN) and Li‐O(PEGDA) appearing near 2.03 Å, suggesting that PEGDA also participates in the inner solvation shell of Li^+^. The broad peaks (4–12 Å) corresponding to Li‐N(SN) and Li‐O(PEGDA) in Figure 3g indicate that both SN and PEGDA are involved in the outer solvation shell of Li^+^. These calculations suggest that PEGDA can penetrate the solvation structure around Li^+^ formed by SN, contributing to the formation of additional free Li^+^. Given that lithium metal can also bond with PEGDA, the introduction of PEGDA is expected to mitigate side reactions between lithium metal and SN, thereby enhancing the interfacial stability of lithium metal batteries.^[^ ^25^ ^]^ Figure 3d presents the electrostatic potential of SN, FEC, and PEGDA, where the nitrogen atoms in SN exhibit a notable negative potential, which are the primary coordinating atoms for lithium ions, but in PEGDA and FEC, the ether oxygen is involved, typically having a more negative potential and participating in coordination.^[^ ^12^, ^26^ ^]^ This finding is consistent with the results of MD simulations.

**Figure 4:**
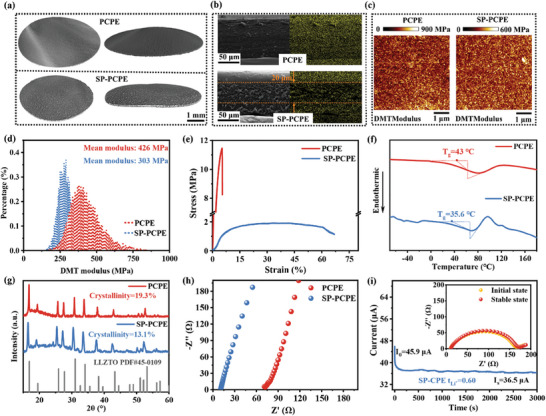
Relevant test analyses for PCPE and SP‐PCPE: a)3D tomography image of PCPE and SP‐PCPE constructed by an X‐ray microscope; b)SEM cross section of CPE and SP‐PCPE surfaces; c)AFM modulus distribution of the substrate surface; d)The modulus histogram in Figure 4c; e) Stress‐strain curves; f) DSC profiles; g) XRD patterns; h) EIS impedance tests; i) Test for Li^+^ transference number of the SP‐PCPE matrix.

The SN within SP will exert a strong plasticization effect on the PCPE matrix. The plasticization effect of SP on PCPE was analyzed by X‐ray microscope, SEM and AFM. Three‐dimensional tomography images (**Figure** **4**a) show that the surface of PCPE is flat, while that SP‐PCPE is homogeneously etched by plasticization effect of SP.^[^ ^27^ ^]^ As depicted in Figure 4b and Figure S5a (Supporting Information), the pristine CPE matrix shows a dense morphology with uniform distribution of F and Zr elements, indicating the uniform distribution of lithium salt and LLZTO in the PCPE matrix. When SP is applied to the PCPE surface, SP can partially diffuse into the SP‐PCPE to form an interfacial layer with a depth of ≈20 µm (Figure 4b). Figure 4b shows that the F element has a stronger signal on the SP‐PCPE surface, while the Zr element is uniformly distributed in PCPE (Figure S5b, Supporting Information). SP can penetrate into the PCPE to a certain extent, comes from that the SN in SP can play the role of plasticizing the PCPE matrix. The plasticizing effect would reduce the modulus of the SP‐PCPE and improve its flexibility. The microscopic mechanical properties of PCPE and SP‐PCPE surfaces were tested by using AFM (Figure 4c). The AFM results shows that the modulus of the SP‐PCPE matrix surface is mainly distributed in the range of 0–600 MPa, lower than that of the PCPE (0–900 MPa). According to AFM results, the calculated average Young's modulus is shown in Figure 4d, which shows a lower modulus of 303 MPa for SP‐PCPE in comparison to that for CEP (426 MPa).

The characterization of tensile strength and strain were conducted to evaluate the mechanical properties of PCPE and SP‐PCPE. As shown in Figure 4e, the PCPE has a tensile strength of 11.5 MPa and a strain of only 5%, indicating a high degree of rigidity. In contrast, the SP‐PCPE after interfacial eutectic polymerization has a reduced tensile strength of 1.91 MPa, and its strain dramatically increases to 67%, which is better than SA‐PCPE (Figure S4b, Supporting Information) and demonstrates a significant macroscopic flexibility in the electrolyte matrix. The stress‐strain results confirm that SN within SP can plasticize the PCPE matrix, which effectively promotes polymer chain segmental motion and enhances ionic conductivity.^[^ ^11a^ ^]^ The DSC results in Figure 4f show that the glass transition temperature of SP‐PCPE is 7.2 °C, which is lower than that of PCPE and is beneficial for ionic transporting.^[^ ^28^ ^]^ X‐ray diffraction (XRD) analysis is then carried out to investigate the influence of SP on the crystalline structure PAN‐based composite polymer electrolyte. The crystallinity of PCPE (19.3%) is calculated from the XRD patterns (Figure 4g; Figure S6, Supporting Information). After applying the SP, the resulting polymer electrolyte shows a decreased crystallinity of 13.1%, which is beneficial for polymer chain segmental motion and Li^+^ transport.^[^ ^29^ ^]^ The EIS impedance of Figure 4h visualizes the intrinsic impedance of PCPE and SP‐PCPE. The ionic conductivity of SP‐PCPE is calculated to be 4.47 × 10^−4^ S cm^−1^, higher than that of CPE (6.54 × 10^−5^ S cm^−1^).

The applying of SP also has a positive effect on accelerating the Li^+^ movement. As shown in Figure S7 (Supporting Information), the transference number of PCPE is only 0.2, while that of SP‐PCPE increases to 0.6 (Figure 4i). This is primarily because the SN provides an effective plasticizing effect on SP‐PCPE, and creates a substantial number of amorphous regions for Li^+^ movement, thereby facilitating Li^+^ migration. Additionally, the SN at the interface of SP‐PCPE with high electronegative N atoms can effectively suppress the accumulation of anions at the interface, resulting in reducing polarization in the SP‐PCPE symmetric battery and enhancing the lithium‐ion transference number.^[^ ^30^ ^]^ The LSV curves in Figure S8 indicate that the electrochemical window of the unmodified PCPE is as high as 5 V, which is mainly caused by the inherent high oxidation resistance of PAN and LLZTO in the matrix. In contrast, the eutectic polymerization‐modified SP‐PCPE shows a slight decrease in oxidation resistance, but its electrochemical window still reaches 4.9 V, which is compatible with traditional high‐voltage cathodes.

### 2.3 Excellent Electrochemical Performances of SP‐PCPE Based Lithium Solid‐State Batteries

**Figure 5:**
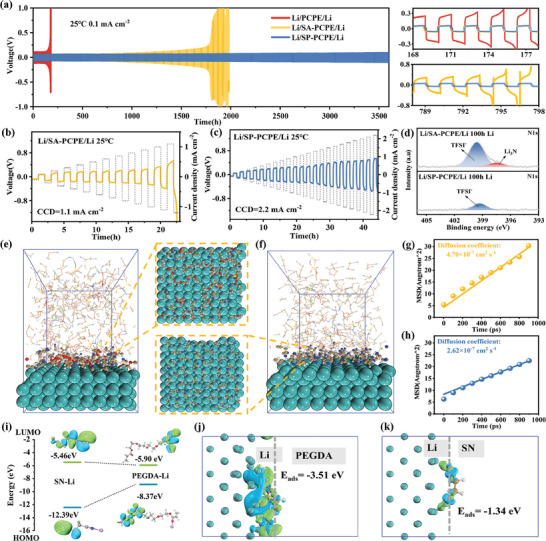
a) Long cycling curves of Li/PCPE/Li, Li/SA‐PCPE/Li, and Li/SP‐PCPE/Li symmetric batteries at a current density of 0.1 mA cm^−2^; b) CCD tests for Li/SA‐PCPE/Li, c) Li/SP‐PCPE/Li batteries; d) XPS spectra of N1s on the Li metal surface after 100 h of cycling at 0.1 mA cm^−2^ for Li/SA‐PCPE/Li and Li/SP‐PCPE/Li symmetric batteries; e) MD simulation of the SP system at the Li metal interface; f) MD simulation of the SA system at the Li metal interface; g) Calculation of MSD for the SA system; h) Calculation of MSD for the SP system; i)HOMO and LUMO for SN‐Li and PEGDA‐Li; j) DFT results of adsorption energy of PEGDA on Li metal; k) DFT results of adsorption energy of SN on Li metal.

To demonstrate electrochemical stability of SP‐PCPE towards lithium metal, Li/PCPE/Li, Li/SA‐PCPE/Li and Li/SP‐PCPE/Li symmetric batteries were assembled, and the Li/SP‐PCPE/Li symmetric battery can stably cycle for as long as 3600 h shown in **Figure** **5**a, with the polarization potential remaining at a low level. The enhanced cycling stability can be attributed to the following two reasons: 1) the high ionic conductivity and Li^+^ transference number of SP‐PCPE lead to promoting Li^+^ transport and reduce polarization potential; 2) the PEGDA anchors SN at the interface of SP‐PCPE, and the anchoring effect makes SN difficult to freely react with Li metal through diffusion, resulting in the constraint of side reactions between SN and Li metal.

To investigate the performance of the SP‐PCPE matrix under high current density, the critical current density (CCD) of Li‐Li symmetric batteries with different electrolyte systems was tested. As shown in Figure S9 (Supporting Information), the PCPE symmetric battery exhibits high polarization potentials at various current densities. The battery's polarization potential of PCPE symmetric battery is up to 0.6 V at the current density of 0.4 mA cm^−2^, then the battery collapses with current density increases to 0.5 mA cm^−2^. The SP‐PCPE battery system shows a higher CCD (1.1 mA cm^−2^) than that of PCPE symmetric battery (Figure 5b), due to the improved ionic conductivity and interfacial contact provided by interfacial plasticization.^[^ ^11a^ ^]^ However, SP‐PCPE exhibits the best lithium stability with the lowest polarization potential of <0.5 V at 2.2 mA cm^−2^, as shown in Figure 5c, demonstrating the excellent tolerance to high currents. As shown in Figure S10 (Supporting Information), Li/SP‐PCPE/Li symmetric batteries can stably cycle for over 300 h at 0.5 mA cm^−2^, further demonstrating the outstanding chemical stability towards Li.^[^ ^31^ ^]^

To further explain the stable cycling of Li–Li batteries, Li metal was separated from both Li/SA‐PCPE/Li and Li/SP‐PCPE/Li batteries after 100 h cycle at 0.1 mA cm^−2^, and the surface composition of the Li metal was analyzed by XPS (Figure S11, Supporting Information). As shown in Figure S11a,d (Supporting Information), the peak at 684.7 eV in F1s is attributed to LiF.^[^ ^32^ ^]^ LiF has a lower adsorption energy with SN, which can suppress the side reactions between SN and Li metal to some extent.^[^ ^16^ ^]^ The peaks located at 686.6 eV in F1s corresponding to the B‐F bonds of ionically conductive Li_x_BO_y_F_z_. B‐F bonds can effectively promote Li^+^ transport at the interface.^[^ ^8^, ^13^ ^]^ The C 1s spectra in Figure S11c,f (Supporting Information) show peaks corresponding to C─C, C─O, and RCO_3_Li at 284.9, 286.5, and 289.9 eV, respectively, which are common components in the SEI.^[^ ^33^ ^]^ Notably, the oxygen‐containing C─O and RCO_3_Li peaks of SP‐PCPE are much stronger than that of CPE, primarily due to the decomposition of PEGDA on Li.^[^ ^33b^ ^]^ Thus, the PEGDA at the SP‐PCPE interface preferentially adsorbs and decomposes on the Li metal, which would further suppress side reactions between SN and Li metal.

The analysis of XPS was further conducted to examine the side reaction in the interface of Li/SA‐CPE/Li and Li/SP‐PCPE/Li. As shown in Figure 5d, for both systems, a peak of TFSI^−^ is detected at 399.5 eV, which originates from the LiTFSI.^[^ ^34^ ^]^ A peak at 398.4 eV belongs to Li_3_N is clearly observed in the spectra of Li/SP‐PCPE/Li, which indicates the occurrence of side reaction between SN and Li metal at the interface.^[^ ^13a,b^ ^]^ However, in the SP‐PCPE system, the N 1s peak at 398.4 eV is no longer observable, suggesting that the 3D network formed in polymerized PEGDA can prevent the side reactions between SN and Li metal. This is because PEGDA at the SP‐PCPE interface effectively anchors SN, and inhibits its diffusion towards the Li metal interface, leading to the suppression of side reactions between them.

These experimental results are going to be proven consistent with the trends observed from subsequent theoretical calculations. Figure 5e presents molecular dynamics simulations of the electrolyte systems' interface with lithium metal, indicating that in the SP‐PCPE, PEGDA, and anions more readily adsorb onto the surface of lithium metal compared to the SA system, where a significant distribution of SN on the lithium metal surface may lead to severe side reactions with lithium metal. Molecular dynamics simulations were utilized to calculate the diffusion coefficients of SN in both the SA and SP systems. As shown in Figure 5g,h, the diffusion coefficients of SN in the SA and SP systems are 4.70 × 10^−7^ cm^2^ s^−1^ and 2.62 × 10^−7^ cm^2^ s^−1^, respectively, suggesting that SN exhibits a significantly lower diffusion coefficient in SP than that in SA. Figure 5k displays the adsorption energy between SN and lithium metal, calculated by using DFT, to be −1.34 eV, indicating that SN can be adsorbed onto the surface of lithium metal and undergo charge transfer, leading to side reactions between lithium metal and SN. For the adsorption calculation between PEGDA and lithium metal, as depicted in Figure 5j, electron‐deficient regions (blue) are primarily located on the surface of lithium metal, while electron‐rich regions (green) are predominantly concentrated on PEGDA molecules. The calculated adsorption energy between PEGDA and lithium is −3.51 eV, significantly lower than that between SN and lithium metal (−1.34 eV), indicating a high propensity for charge transfer between PEGDA and lithium. The LUMO and HOMO energy levels of SN‐Li and PEGDA‐Li were calculated and presented in Figure 5i, with the LUMO energy level of PEGDA‐Li (−5.90 eV) being lower than that of SN‐Li (−5.46 eV), suggesting that PEGDA is more likely to undergo reduction reactions with lithium metal in the Li/SP‐PCPE/Li system.^[^ ^35^ ^]^ The frontier orbitals of different molecules with lithium salts (Figure S12, Supporting Information) indicate that LiDFOB is more prone to oxidative‐reduction decomposition than LiTFSI, forming a solid electrolyte interphase containing B and F element, which benefits the electrode. In summary, PEGDA can preferentially adsorb onto the lithium metal surface, forming a steric hindrance effect that prevents SN from adsorbing onto the lithium metal surface. Furthermore, PEGDA can interact with SN to suppress its free shuttling, and with its lower LUMO energy level, it can be preferentially reduced with FEC to form a stable SEI membrane, leading to the effective constraint of side reactions between SN and lithium metal.

**Figure 6:**
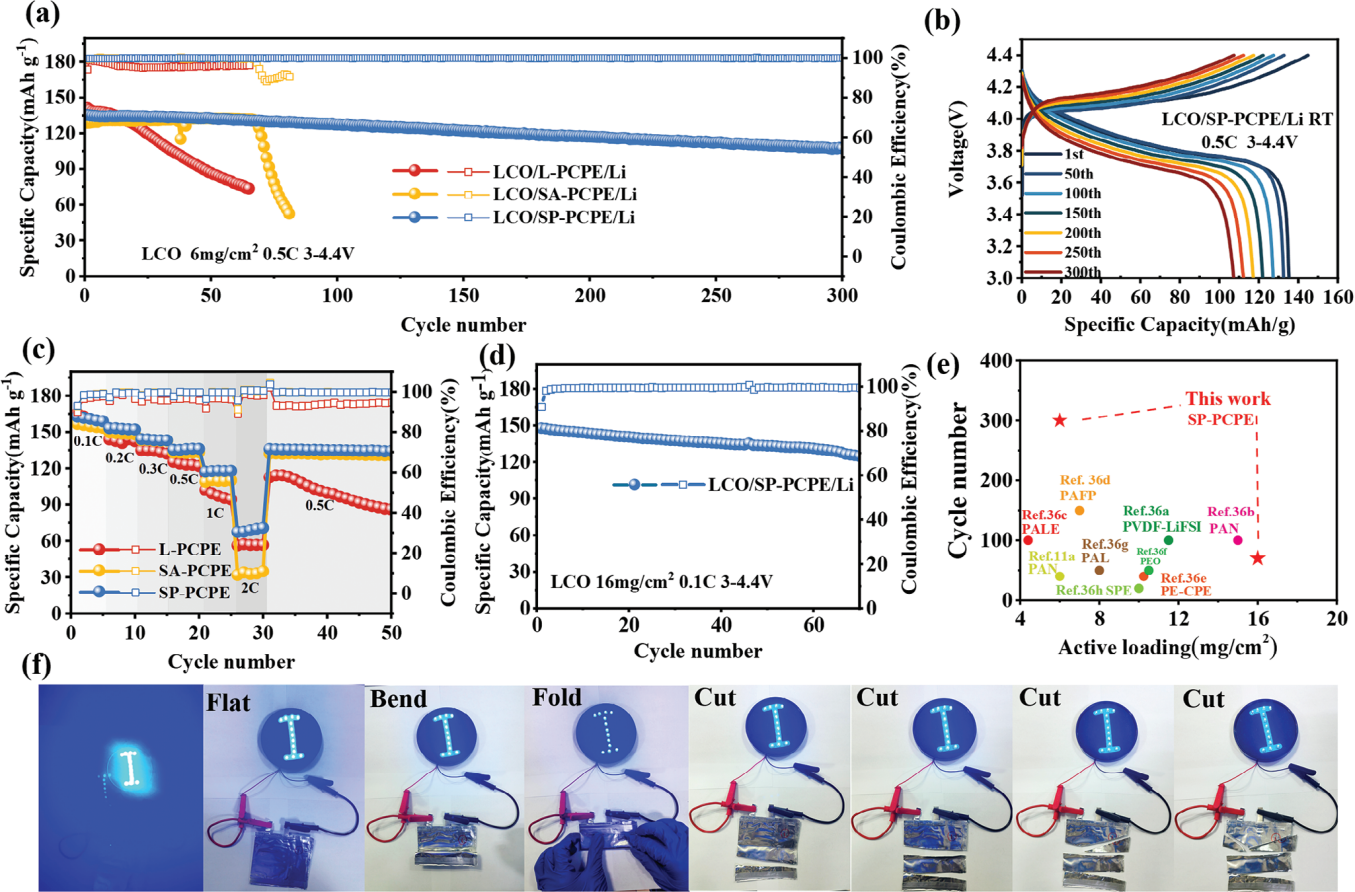
a) Long cycling curves of LCO/L‐PCPE/Li, LCO/SA‐PCPE/Li, and LCO/SP‐PCPE/Li at a rate of 0.5 C; b) Charge‐discharge curves of the LCO/SP‐PCPE/Li battery during cycling at 0.5 C; c) Rate performance of LCO/L‐PCPE/Li, LCO/SA‐PCPE/Li, and LCO/SP‐PCPE/Li batteries from 0.1 C to 2 C; d) Cycling performance for high mass loading at 0.1 C (16 mg cm^−2^) LCO /SP‐PCPE/Li battery; e) Comparative analysis of Electrochemical performance of solid‐state lithium batteries with mass loading higher than 4 mg cm^−2^; f) Photographs of the LCO/SP‐PCPE/Li pouch cell lighting an LED in various states, including flat, bent, fold, and cut.

To verify practical applications of SP‐PCPE, solid‐state batteries with high‐loading cathodes were assembled. As shown in Figure S13 (Supporting Information), interface impedance tests were conducted on full cells using different electrolyte and high loading (6 mg cm^−2^) LCO. The interface impedance of the PCPE battery without any interfacial treatment is higher than 1000 Ω, while the interface impedances of full batteries are measured to be 90, 98, and 80 Ω, respectively, for batteries with the addition of liquid electrolytes, SA, and SP. The findings show that has the greatest interfacial improvement can be achieved for SP‐PCPE in comparison to other methods when matched with high‐loaded electrodes. **Figure** **6**a displays the cycling performance of the LCO/electrolyte/Li battery systems at a voltage range of 3.0–4.4 V and a rate of 0.5 C. Typically, without any interfacial treatments, the full battery of LCO/PCPE/Li exhibits low capacity and poor cycling performance, which cannot meet the practical application requirements.^[^ ^8a^ ^]^ In practice, a small amount of liquid electrolyte is commonly used to improve the interface of PCPE. As shown in Figure 6a, for the liquid electrolyte‐modified PCPE (L‐PCPE), the battery capacity is fully utilized at the beginning of the cycle, but as the cycling progresses, the capacity continuously decays due to the inevitable consumption of the liquid electrolyte, and the capacity retention after 65 cycles is only 52%. Our previous studies have shown that when matched with low‐loading LFP cathodes, SA‐PCPE modified with SA exhibits excellent cycling stability.^[^ ^11a^ ^]^ However, the LCO/SA‐PCPE/Li cell can cycle stably only for the first 67 cycles, and then the capacity decreases dramatically (Figure 6a). Based on the above analysis, this may be due to side reactions between SN and Li metal, and the poor wettability of SA on high load electrodes. Significantly, for the SP‐PCPE, benefiting from the excellent infiltration of SP on the highly loaded cathode and the anchoring effect of polymerized PEGDA on SN, the initial discharge capacity of LCO/SP‐PCPE/Li is 135.3 mAh g^−1^, with a capacity retention of 94.1% after 100 cycles, and even after 300 cycles, the capacity retention remains a high value of 80.0%. Figure 6b shows the charge/discharge curves of the LCO/SP‐PCPE/Li battery at 0.5 C. Because of the excellent ionic conductivity of SP‐PCPE and the construction of a stable and intimate interface, LCO/SP‐PCPE/Li batteries exhibit a small polarization and high capacity retention during cycling. Figure 6c presents the rate performance of LCO/L‐PCPE/Li, LCO/SA‐PCPE/Li, and LCO/SP‐PCPE/Li batteries. From a rate of 0.1 C to 2 C, the LCO/SP‐PCPE/Li battery system can provide specific capacities of 162.7, 135.9, and 70.8 mAh g^−1^, respectively. When the rate is returned to 0.5 C, the capacity can also recover to 136.0 mAh g^−1^, demonstrating the superior reversibility compared to the LCO/L‐PCPE/Li and LCO/SA‐CPE/Li battery systems.

Batteries in practical applications often need to be equipped with ultra‐high loading electrodes to increase the energy density. To test the compatibility between the SP‐PCPE system and ultra‐high loading LCO, batteries were assembled using ultra‐high loading LCO (16 mg cm^−2^) and SP‐PCPE. As shown in Figure 6d, the SP‐PCPE battery system can stably cycle for 70 cycles at a rate of 0.1 C, indicating that the eutectic polymerized SP‐PCPE can effectively match ultra‐high loading cathodes and ensure stable battery cycling. Even paired with NCM811 with higher capacity (4.4 V, 8 mg cm^−2^), the SP‐PCPE battery system can also cycle relatively stably (coulombic efficiency of 99.7%, as shown in Figure S14, Supporting Information), and deliver a capacity of over 2 mAh. As shown in Figure 6e and Table S1 (Supporting Information), benefiting from the high wettability and interfacial stability of the SP‐PCPE, our high‐loading batteries exhibit a significant advantage over the latest literatures on solid‐state batteries with active loading higher than 4 mg cm^−2^,^[^ ^11^, ^36^ ^]^ making our design a viable option for practical applications.

Finally, a LCO/SP‐PCPE/Li pouch cell was assembled to explore the practicality of the SP eutectic polymerization‐modified PCPE, and an optical image of the cell lighting an LED lamp was also presented. As shown in Figure 6f, the pouch cell effectively lit the LED lamp in a flat state, and even under mechanical deformations such as bending and folding, the LED lamp continues to function normally, indicating the pouch cell after interfacial eutectic polymerization exhibited excellent flexibility. Figure 6f also shows that even under extreme conditions of being cut in half or cut into multiple segments, the pouch cell did not short‐circuit and could still continuously light the LED. These results demonstrate that the LCO/SP‐PCPE/Li pouch cell exhibits good safety and reliability, and the method of improving the interface with an SP eutectic electrolyte layer greatly enhances the practicality of PAN‐based CPE when using the high‐loading cathodes.

## 3 Conclusion

In this study, PEGDA and FEC are deliberately combined with SN and LiTFSI/LiDFOB to form a deep eutectic polymeric liquid (SP). Upon polymerization, SP becomes a deep eutectic electrolyte that effectively improves the interface of PAN‐based CPEs. The dilution of SN by PEGDA and FEC enhances the wettability of SP towards thick cathode, and the strong interaction between PEGDA, FEC and SN can create a competitive effect for Li^+^, which promotes the dissociation of lithium salts and facilitates the fast Li^+^ transport. After the eutectic polymerization of SP, PEGDA firmly anchors SN, which effectively suppresses side reactions between SN and Li metal, and ameliorates the contact at the electrolyte/electrode interface. Furthermore, the SN from deep eutectic polymeric interface can act as a plasticizer to reduce the crystallinity of PAN in the matrix, and enhance the ionic transport of the PCPE. As a result, the SP enhanced PAN‐based CPE exhibits excellent Li^+^ conduction characteristics with a high ionic conductivity of 4.47 × 10^−4^ S cm^−1^ and high lithium transference number of 0.60. SP‐PCPEs also present an outstanding lithium stability, which can be verified by the long stable cycling of Li‐Li battery for 3600 h at 0.1 mA cm^−2^ and a critical current density of 2.2 mA cm^−2^. The LCO/SP‐PCPE/Li batteries with a higher loading of 6 mg cm^−2^ at 3–4.4 V can also achieve a steady cycling for over 300 cycles at 0.5 C. Even with an ultra‐high loading of 16 mg cm^−2^, the LCO/SP‐PCPE/Li batteries are capable of stably cycling for 70 cycles at 0.1 C. This approach is of significant importance for the practical application of PCPE.

## Conflict of Interest

The authors declare no conflict of interest.

## Supporting information



Supporting Information

## Data Availability

The data that support the findings of this study are available on request from the corresponding author. The data are not publicly available due to privacy or ethical restrictions.
