# Supplementary material for: In Situ Constructing Robust Interface by Deep Eutectic Polymeric Electrolyte Enables High Performance Lithium Metal Batteries with High‐Loading Cathode
Source: Adv Sci (Weinh). 2024 Oct 28;11(47):2411421. doi: 10.1002/advs.202411421 (PMC11653719; doi:10.1002/advs.202411421)
Supplement: Supplementary file 1 — Supporting Information [file ADVS-11-2411421-s001.docx]

Supporting Information

**In-situ Constructing Robust Interface by Deep Eutectic Polymeric Electrolyte Enables High Performance Lithium Metal Batteries with High-Loading Cathode**

*Zixuan Fang, Ming Zhang, Zhihao Zhang, Jitao Li, Haofeng Peng, Jintian Wu^*^, Haiping Zhou, Ziqiang Xu^*^, Mengqiang Wu^*^*

Z.X. Fang, M. Zhang^.^, Z. H. Zhang, H.F Peng, H. P. Zhou, Z. Q. Xu, M. Q. Wu

*School of Materials and Energy, University of Electronic Science and Technology of China, Chengdu 611731, Sichuan, China*

Email: nanterxu@uestc.edu.cn (Z. Xu); mwu@uestc.edu.cn (M. Wu)

J.T. Li

*School of Precision Instruments and OptoElectronics Engineering, Tianjin University, Tianjin 300072, China*

Z. Q. Xu, M. Q. Wu

*Yangtze Delta Region Institute (HuZhou), University of Electronic Science and Technology of China, Huzhou 313001, Zhejiang, China*

J. T. Wu

*School of Chemical Engineering, Sichuan University of Science & Engineering, Zigong 643000, China*

Email:: wjt@suse.edu.cn (J. Wu)

**Experimental Section/Methods**

*Materials*: Li_6.4_La_3_Zr_1.4_Ta_0.6_O_12_ (LLZTO) was prepared by conventional solid phase sintering method, details of which can be found in our previous work.^[1]^ Both PAN (Mw~150,000, Macklin) and LiTFSI (99%, Aladdin) were dried at 100°C for 24 h prior to use. LiDFOB and FEC were sourced from Suzhou Duo Duo Chemical Technology Co., LTD. Both SN (99%) and PEGDA (Mn~400) were purchased from Aladdin. N, N‑dimethylformamide (DMF) was obtained from Sinopharm. LB002 (1M LiPF_6_ in EC: DMC: EMC=1:1:1 Vol%) was bought from Suzhou DuoDuo Chemical Technology Co., LTD as a conventional liquid electrolyte (LE).

*Preparation of PAN-based CPE(PCPE)*: PAN (1 g) and LiTFSI (1 g) were dissolved in 10 mL of DMF and stirred at 60 °C for 3 h to produce a homogeneous solution. LLZTO (5 wt% of the total amount of PAN and LiTFSI) was added to DMF and sonicated for 30 min, then stirred magnetically for 3 h to completely disperse LLZTO and obtain a stable suspension. The above suspension was stirred with the PAN/LiTFSI/DMF mixture for 8 h to obtain a homogeneous slurry with proper viscosity. After degassing, the mixed slurry was cast onto a clean glass plate using a doctor’s blade. Finally, the PAN-based composite electrolyte containing LLZTO was dried in a vacuum oven at 40 °C for 24 h and then at 80 °C for 24 h to further remove residual solvents.The average thickness of the PAN-based CPE film was about 100 μm.

*Preparation of SP and SA and equipment solid-state cell*: At 70°C, LiTFSI and LiDFOB were dissolved in molten succinonitrile (SN) with a mass ratio of SN:LiTFSI:LiDFOB=50:3:2 and only 5% vol FEC to prepare a succinonitrile-based plasticizing curing agent (SA). Subsequently, 1.5, 3, 6, 12, and 24 wt% of PEGDA were introduced into the aforementioned SA to obtain eutectic polymeric liquids (SP, an additional 5 vol% was also added) with different ratios. The polymerization monomer AIBN (1 wt% of PEGDA) was added as a thermal initiator to explore the minimum amount of PEGDA required for polymerization. SP was drop-casted on both sides of the PCPE at a rate of 3.5 μL cm^-2^, and the PCPE post-eutectic polymerization (eutectic electrolyte SP modified CPE) was referred to as SP-PCPE. Li-Li symmetric batteries were assembled using the electrolyte. The full battery composition included LCO or NCM811 cathodes, Li anodes, and electrolytes. After assembling the SP-PCPE system batteries, they were placed in an oven at 60°C for 30 minutes to ensure complete polymerization of the SP at the interface. The cathodes were prepared using conventional processes, mixing 90 wt% active material (LiCoO_2_ or NCM811), 2.5 wt% Super P, 2.5 wt% carbon nanotubes, and 5 wt% PVDF, and then coated onto aluminum foil. Two different loadings of LiCoO_2_ were selected, 6 mg cm^-2^ and 16 mg cm^-2^, and the NCM811 loading was 7~8 mg cm^-2^. LE (L-PCPE) and SP (10 vol% FEC, SP-PCPE) were drop-casted on both sides of the electrodes at a rate of 3.5 μL cm^-2^ as comparative samples for the other two groups of batteries.

*Material characterization:* Contact angle testing was performed using Dataphysics DCAY21.The modulus distribution of the composite film surface before and after modification was analyzed using atomic force microscopy (AFM, Bruker Dimension Icon). The crystallinity of the different samples was characterized by X-ray diffraction (XRD, Bruker-D8 Advanced X-ray Diffractometer Cu Kα radiation: λ = 1.5406 Å). The 3D tomography images were constructed by an X-ray microscope (Zeiss Xradia 520 versa). Field-emission scanning electron microscopy (FE-SEM, JSM-7500F, JEOL) was used to characterize the morphology of the matrix before and after modification. Fourier transform infrared spectroscopy (FTIR, Thermo Scientific Nicolet 6700) was used to characterize the polymerization of PEGDA and the interaction between PEGDA and SN. Differential scanning calorimetry (DSC, Netzsch DSC 200 F3) was used to characterize the interaction between PEGDA and SN as well as the glass transition temperatures of different electrolyte matrices. Stress-strain curves (NSTRON 5982) of the membranes were measured to assess the mechanical properties. X-ray photoelectron spectroscopy (XPS, Thermo Scientific K-Alpha^+^) was used to obtain the surface composition of the Li anode.

*Electrochemical characterization:* The crystallinity of SN and PAN in different materials was roughly calculated by Hinrichen’s method by the following equation:^[2]^

| $\text{C=}\frac{\text{S}_{\text{c}}}{\text{S}_{\text{c}}\text{+}\text{S}_{\text{a}}}$ | (1) |
| --- | --- |

Where S_c_ represents the area under the crystalline diffraction peaks and S_a_ is the area of the amorphous zone.

The EIS measurements of symmetric stainless steel (SS)/electrolyte/SS cells were used to measure ionic conductivities in the frequency range from 10^-1^ to 10^6^ Hz. 50 µL of SA was injected into polyimide (PI, 22 µm, Jiangxi Advanced Nanofiber S&T Co., Ltd.) membrane to measure the ion conductivity of SA. The same amount of SP was also injected into the PI membrane and the ionic conductivity of SP was measured after thermal polymerization at 60 °C. All the ionic conductivity can be calculated by the formula (3) as follow:

| $\text{σ=}\frac{\text{L}}{\text{RS}}$ | (2) |
| --- | --- |

Where L is the thickness of the electrolyte film, R and S are the total resistance of the electrolyte and the contact area of the electrolyte with the steels. The linear sweep voltammogram tests of Li/electrolyte/SS cells were performed over the potential range of 2 ~ 6 V (vs Li^+^/Li) to evaluate the electrochemical stable window of the electrolyte. The Li^+^ transference number (t_Li+_) was estimated by AC impedance and DC polarization with Li-Li symmetric cells and calculated by the Bruce Vincent Evans Equation (4)

| $\text{t}_{\text{Li}^{\text{+}}}\text{=}\frac{\text{I}_{\text{s}}\text{(}\text{∆V-}\text{I}_{\text{0}}\text{R}_{\text{0}}\text{)}}{\text{I}_{\text{0}}\text{(}\text{∆V-}\text{I}_{\text{s}}\text{R}_{\text{s}}\text{)}}$ | (3) |
| --- | --- |

Where *Is* and *I_0_* are the steady-state and initial currents, respectively. *R_0_* and *R_s_* are defined as the initial and steady-state interfacial resistances between the electrolyte and electrodes, and *ΔV* is the polarization voltage, which was used as 10 mV in this experiment. These tests and subsequent full battery impedance tests were performed on a CHI660E electrochemical workstation. All the full cells and Li symmetric cells were carried out on Wuhan Land CT2001A battery testing system at room temperature.

*Theoretical calculation Methods:* All molecular dynamics (MD) simulations were performed using the Gromacs 2020 software suite.^[3]^ The simulation systems were constructed to represent solid polymer composite electrolyte (SP) and composite polymer electrolyte (SA) models. The SP model was assembled by uniformly incorporating 14 LiTFSI, 18 LiDFOB, 811 sucrose nonaflate (SN), 45 fluoroethylene carbonate (FEC), and 52 polyethylene glycol diacrylate (PEGDA) molecules within a periodic unit cell. The SA model was similarly constructed with 14 LiTFSI, 18 LiDFOB, and 832 SN molecules, along with 7 FEC molecules. The generation of these systems was facilitated using the Packmol software.^[4]^

For the simulation of polymeric and organic components, the Generalized Amber Force Field (GAFF) was employed,^[5]^ while the Merz-Kollman Optimized Potentials for Liquids (OPC3) force field parameters were utilized to describe the metal ions . The atomic charges and force field parameters for the electrolyte components were derived using the Multiwfn program and sobtop by fitting to the electrostatic potential of the molecular structures optimized at the B3LYP/def2TZVP level of theory within the Orca 6.0 computational chemistry package.^[6]^

Van der Waals interactions were modeled using the Lennard Jones (LJ) potential with a cutoff distance of 1.2 nm. Long-range electrostatic interactions were accounted for using the particle-mesh Ewald method.^[7]^ Initial configurations were subjected to energy minimization using the steepest descent algorithm to ensure a stable starting geometry.

Subsequent to minimization, the systems were equilibrated and subjected to MD simulations at a temperature of 298.15 K and a pressure of 1 bar. These conditions were maintained using the V-rescale thermostat^[8]^ and the Berendsen barostat [9],^[9]^ respectively. The MD trajectories were integrated using the leapfrog algorithm with a time step of 1 femtosecond, resulting in a total simulation time of 10 ns for each system.

Throughout the simulations, three-dimensional periodic boundary conditions were applied to mimic an infinite system and to prevent edge effects. The simulation parameters and protocols were chosen based on standard practices in MD simulations for electrolyte systems and were validated to ensure the physical relevance of the results obtained.

The intermediate stable region was chosen as the basis for calculating the ionic or molecular diffusion coefficients, as shown in the equations below:

| $\text{MSD}\left( \text{r}^{\text{2}} \right)\text{=6Dt+C}$ | (4) |
| --- | --- |

Where MSD denotes the mean square displacement, t is the time, D represents the diffusion coefficient, and C denotes constant.

The adsorption energy were carried out using spin-polarized methods as implemented in the QUICKSTEP code of the CP2K 2024 package which based on density functional theory. The general gradient approximation(GGA) parametrized by Perdew, Burke, and Ernzerhof(PBE) was used as the exchange-correlation functional. The Kohn–Sham(KS) equations are solved according to the Gaussian and plane wave (GPW) formalism. Grimme’s DFT-D3 correction was adopted to describe the weak van der Waals interaction. The GPW uses Goedecker–Teter–Hutter pseudo potentials to describe the interactions between core and valence electrons, while the valence electron density is represented in terms of Gaussian type orbital (GTO) basis set functions. In particular, we use DZVP-MOLOPT-SR-GTH basis sets for geometry optimization and TZVP-MOLOPT-SR-GTH basis sets for static calculations, and the Brillouin zone integration was sampled using a Monkhorst-Pack special k-point mesh with a resolution of 2π*0.04 was applied. The convergence criterion for the maximum force was set as 5 × 10^−4^ atomic units. The auxiliary PW basis set, which is needed for the efficient solution of the Poisson's equation in reciprocal space, is truncated at 500 Ry. For the slab models, a vacuum layer of at least 15 Å in thickness was introduced for all the surfaces and interfaces, and dipole correction was considered.

For all quantum calculations, the LUMO and HOMO energy levels of the electrolyte components were calculated and the electrostatic potentials of the different components were derived using ORCA 6.0 software based on density functional theory (DFT). Geometry optimization and energy calculations were carried out at B3LYP/def2TZVP.

**Supporting Figures**


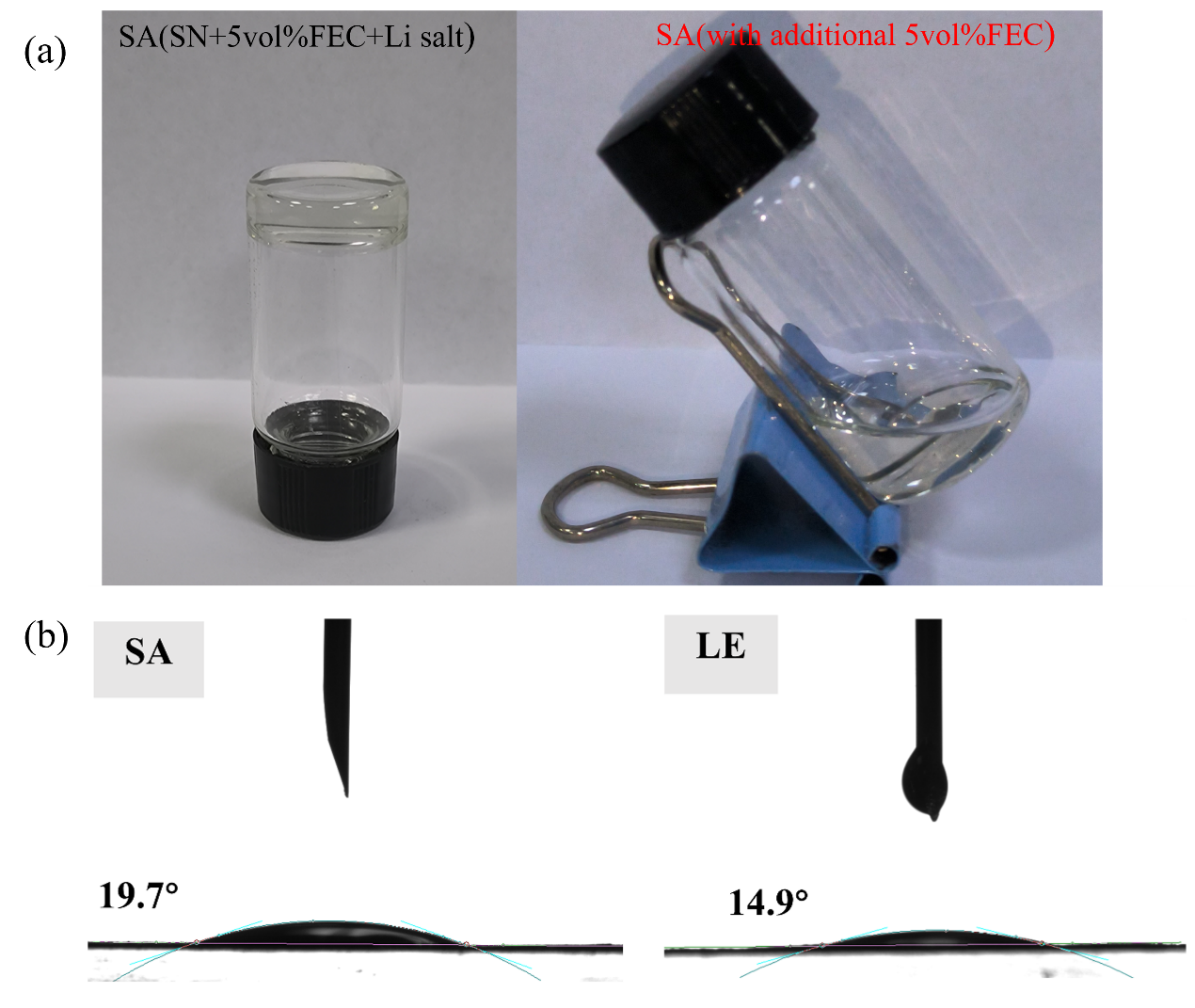


**Figure S1.** a) Optical images of the SA system and the SA system with an additional 5vol% FEC to form a solvent SN+10vol% FEC + LiTFSI/LiDFOB at room temperature; b) Contact angle test of SA as well as LE against LCO.


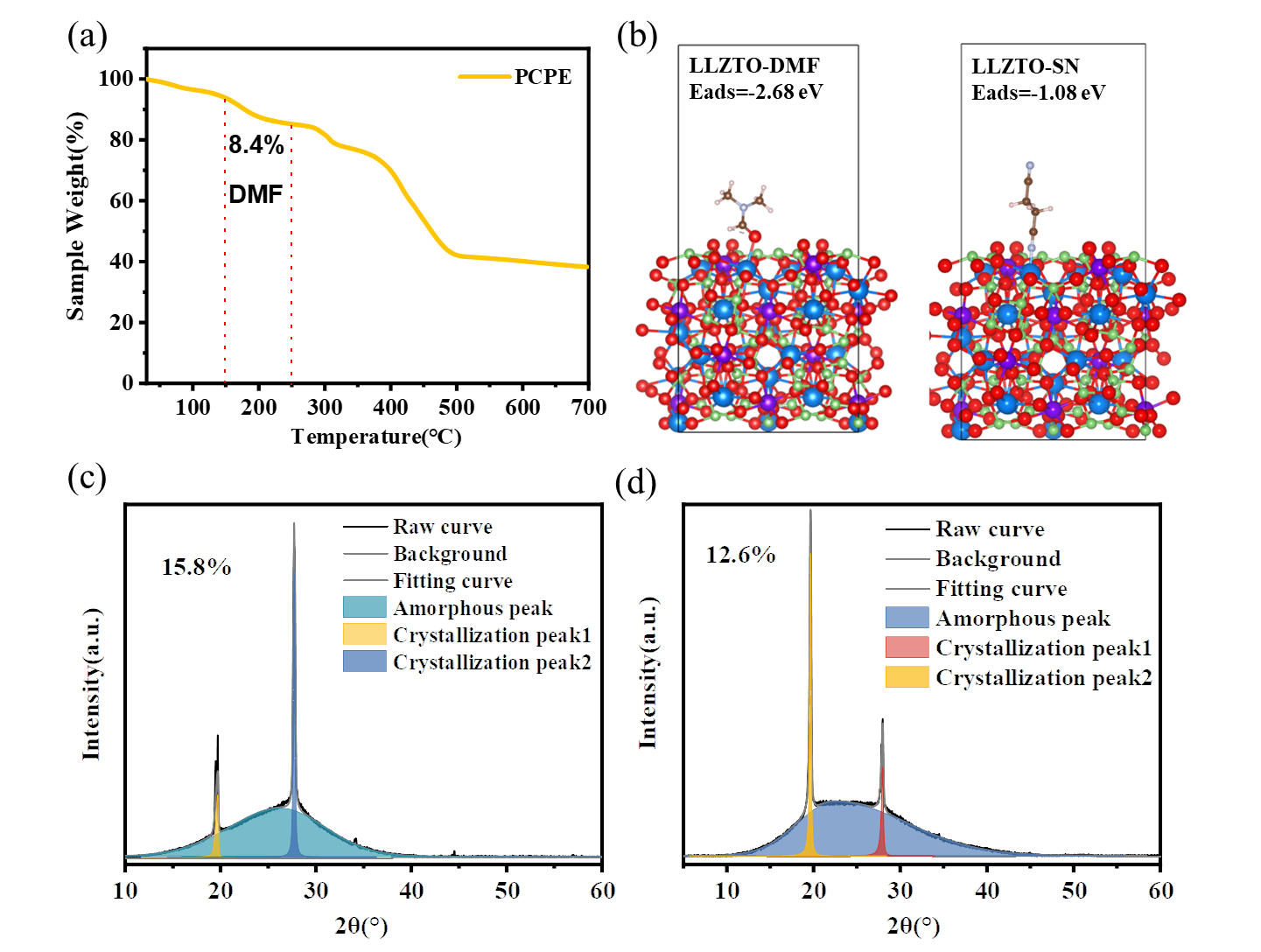


**Figure S2**. a) TGA curves of PCPE, b) DFT calculations of the adsorption energies of LLZTO with SN and DMF; XRD spectra of c) SN, d) SN+PEGDA and their fitted peaks.


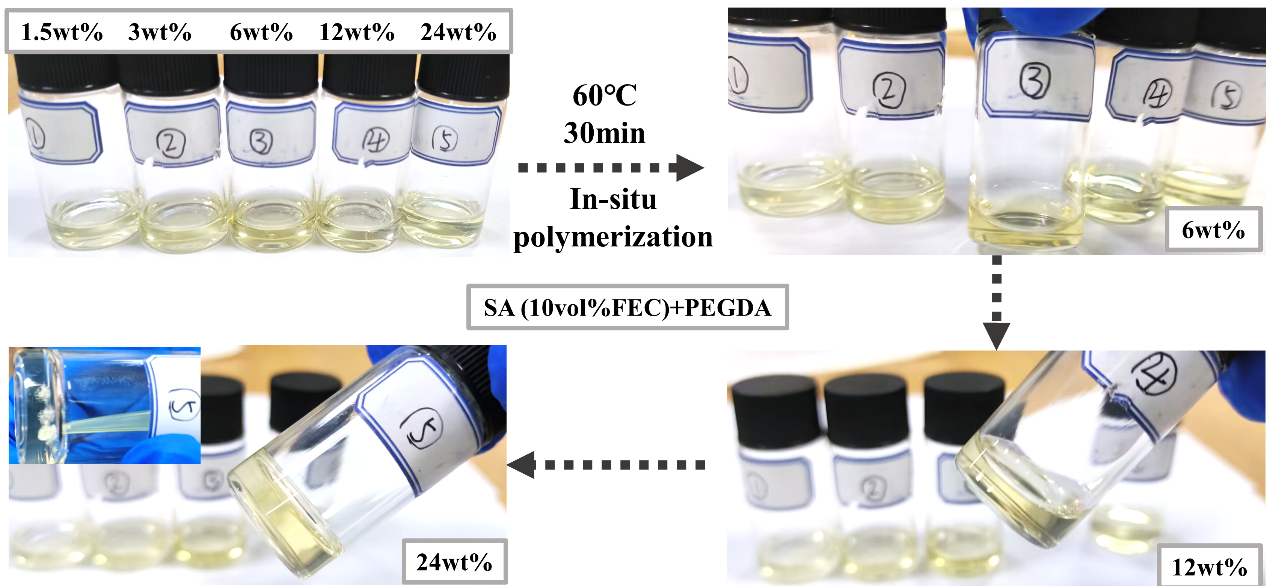


**Figure S3.** Optical pictures of SP polymerized at 60 °C with different PEGDA contents


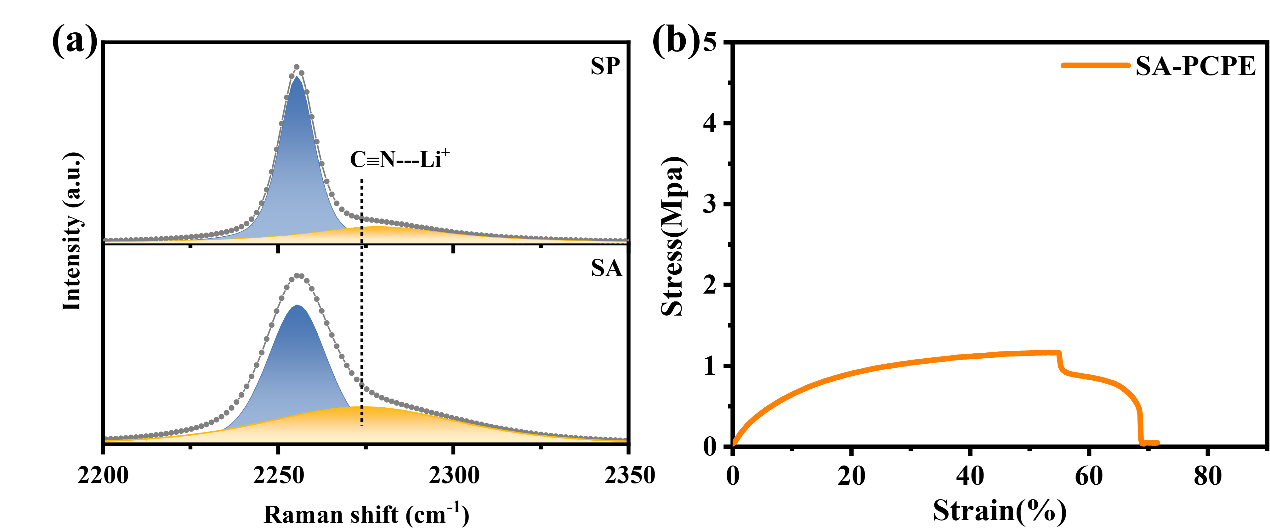


**Figure S4.** a) Raman spectra of SA and SP, and b) the Stress-strain curves of SA-PCPE.


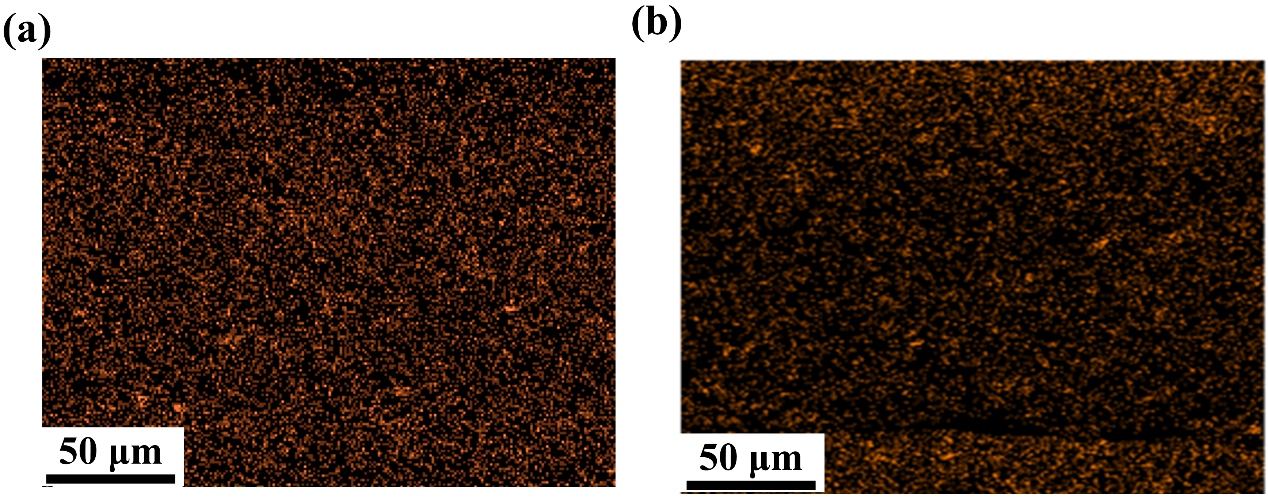


**Figure S5.** EDS pattern of Zr at the interface of PCPE and SP-PCPE


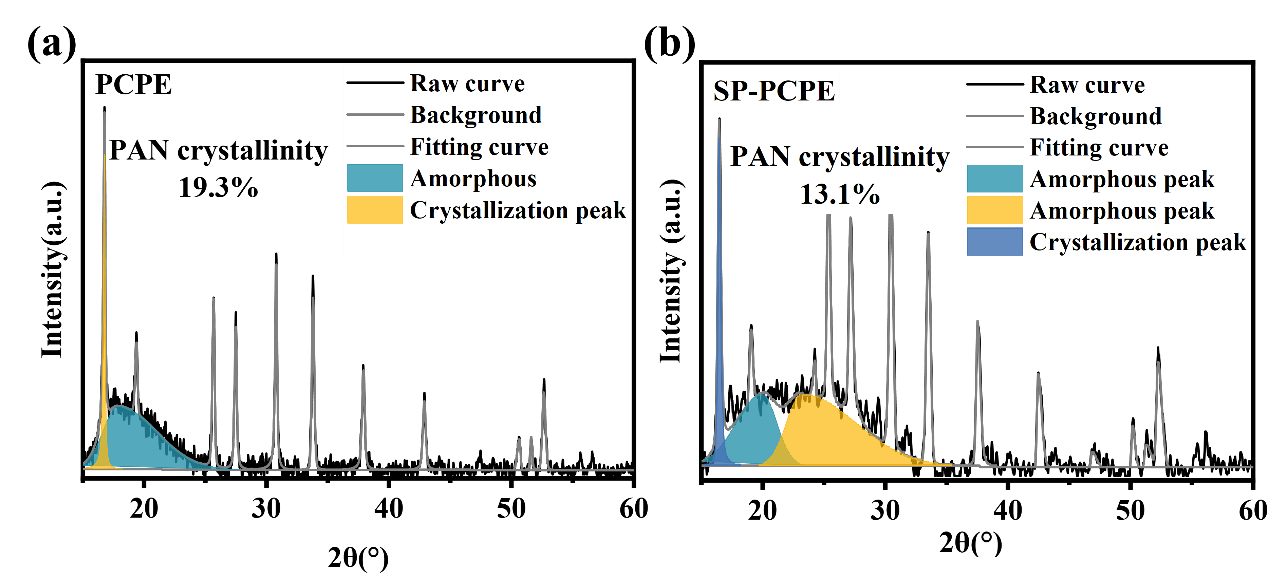


**Figure S6.** XRD spectra of a) PCPE, b) SP-PCPE, and their fitted peaks.


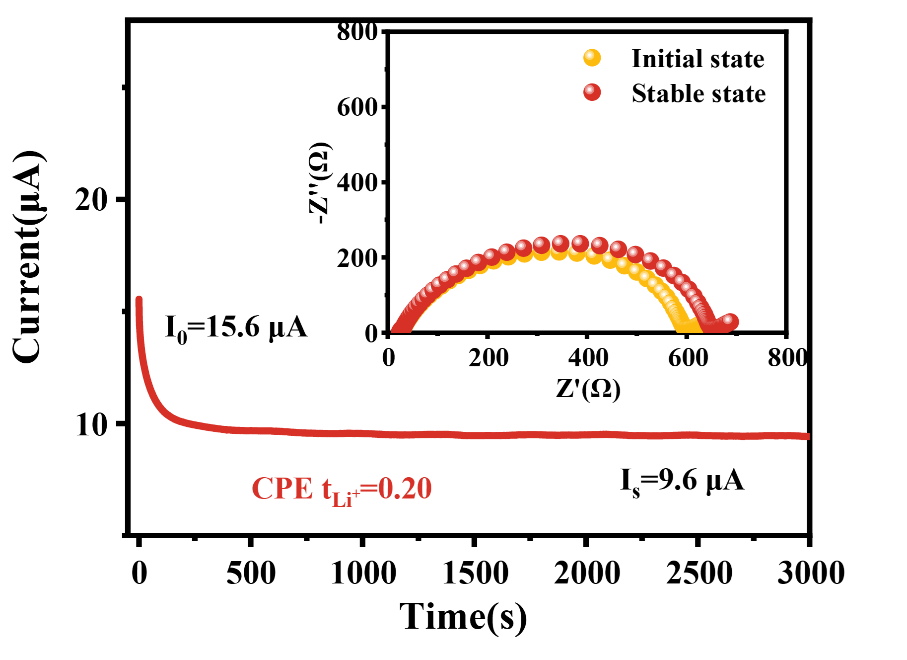


**Figure S7.** Li^+^ transference number for PCPE

**Figure S8.** LSV test for CPE, SP-CPE

**Figure S9.** CCD testing of Li/PCPE/Li batteries

**Figure S10.** Li-Li long cycle diagram of Li-SP-CPE/Li, Li-SP-CPE/Li symmetric cells at 0.5 mA cm^-2^ current density


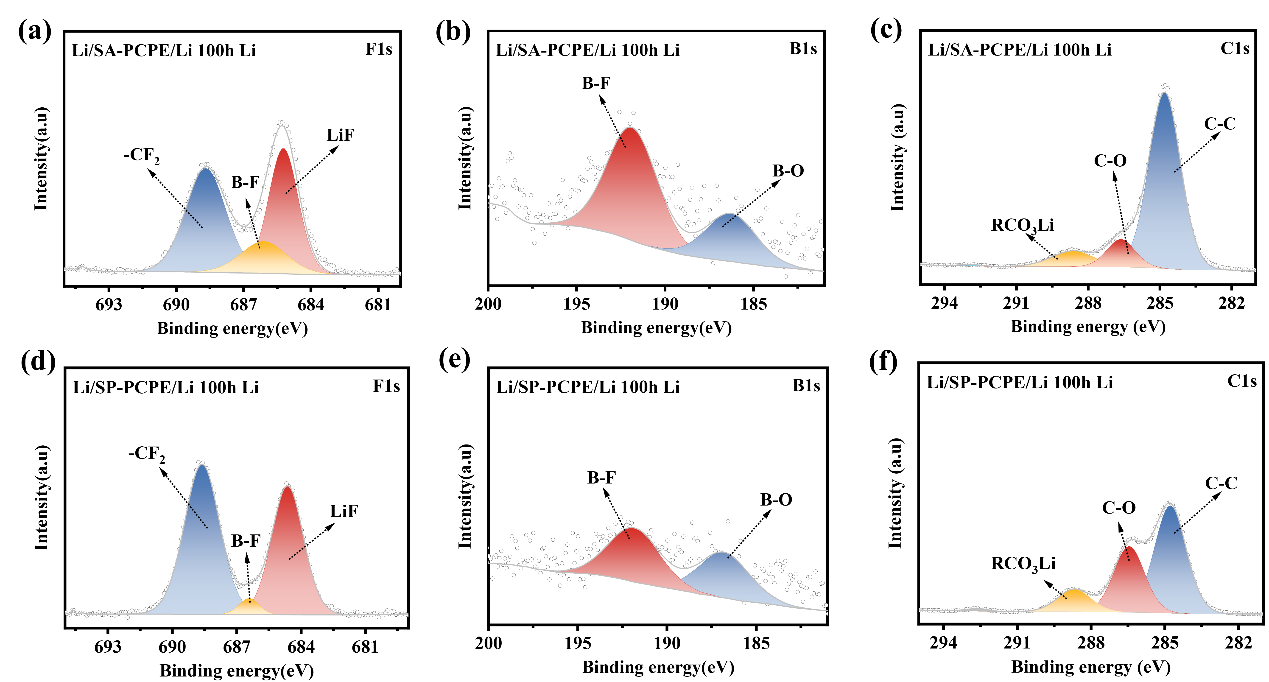


**Figure S11.** XPS patterns of F 1s, B 1s, and C 1s on the Li metal surface of Li/S-CPE/Li and Li/SP-CPE/Li symmetric cells after cycling for 100 h at 0.1 mA cm^-2^ current density.

**Figure S12.** The molecular frontier orbitals of various molecules calculated by Density Functional Theory.

**Figure S13.** EIS impedance spectra of LCO/Li batteries with different interfacial modification methods for PCPE

**Figure S14.** Cycling performance at 0.2 C for high massloading NCM811 (8 mg cm^-2^)/SP-CPE/Li battery

**Table S1.** Comparative analysis of Electrochemical performance of solid-state lithium batteries with mass loading higher than 4 mg/cm^2^

| Cathode | Electrolytes | Temp. | Loading (mg/cm^2^) | Rate (C) | Retention (%) | Ref. |
| --- | --- | --- | --- | --- | --- | --- |
| LiFPO_4_ | PVDF-LiFSI | RT | 11.5 | 0.05 | 40 | ^[10]^ |
| LiCoO_2_ | PAN | RT | 6 | 1 | 95 | ^[11]^ |
| LiCoO_2_ | PAN | RT | 15 | 0.5 | 91 | ^[12]^ |
| LiFPO_4_ | PALE | RT | 4.4 | 0.5 | 73.3 | ^[13]^ |
| LiCoO_2_ | PAFP | RT | 7 | 0.5 | 82 | ^[14]^ |
| LiCoO_2_ | PE-CPE | RT | 10.24 | 0.05 | 81 | ^[15]^ |
| LiFPO_4_ | PEO | RT | 10.5 | 0.1 | 83 | ^[16]^ |
| LiFPO_4_ | PAL | 60℃ | 8 | 0.1 | 83 | ^[17]^ |
| LiFPO_4_ | SPE | RT | 10 | 0.2 | 90 | ^[18]^ |
| LiCoO_2_ | SP-PCPE | RT | 6 | 0.5 | 78 | This work |
| LiCoO_2_ | SP-PCPE | RT | 16 | 0.1 | 80 | This work |

References

[1] Z. Xu, X. Hu, B. Fu, K. Khan, J. Wu, T. Li, H. Zhou, Z. Fang, M. Wu, *J. Materiomics* **2023**, *9*, 651-660.

[2] M. Yu, C. Wang, Y. Bai, Y. Wang, Y. Xu, *Polym. Bull.* **2006**, *57*, 757-763.

[3] H. J. C. Berendsen, D. van der Spoel, R. van Drunen, *Computer Physics Communications* **1995**, *91*, 43-56.

[4] M. G. Martin, J. I. Siepmann, *The Journal of Physical Chemistry B* **1998**, *102*, 2569-2577.

[5] J. Wang, R. M. Wolf, J. W. Caldwell, P. A. Kollman, D. A. Case, *Journal of Computational Chemistry* **2004**, *25*, 1157-1174.

[6] a)F. Neese, *WIREs Computational Molecular Science* **2012**, *2*, 73-78; b)T. Lu, F. Chen, *Journal of Computational Chemistry* **2012**, *33*, 580-592.

[7] T. Darden, D. York, L. Pedersen, *The Journal of chemical physics* **1993**, *98*, 10089-10092.

[8] G. Bussi, D. Donadio, M. Parrinello, *The Journal of Chemical Physics* **2007**, *126*.

[9] H. J. C. Berendsen, J. P. M. Postma, W. F. van Gunsteren, A. DiNola, J. R. Haak, *The Journal of Chemical Physics* **1984**, *81*, 3684-3690.

[10] Q. Kang, Y. Li, Z. Zhuang, D. Wang, C. Zhi, P. Jiang, X. Huang, *J. Energy Chem.* **2022**, *69*, 194-204.

[11] Z. Zhang, M. Zhang, J. Wu, X. Hu, B. Fu, X. Zhang, B. Luo, K. Khan, Z. Fang, Z. Xu, M. Wu, *Small* **2024**, *20*, 2304234.

[12] Y. Chen, S. Liu, S. Cheng, S. Gao, J. Chai, Q. Jiang, Z. Liu, X. Liu, J. Liu, M. Xie, W. Dai, *ACS Appl. Energy Mater.* **2022**, *5*, 3072-3080.

[13] S. Chai, Z. Chang, Y. Zhong, Q. He, Y. Wang, Y. Wan, M. Feng, Y. Hu, W. Li, W. Wei, A. Pan, *Adv. Funct. Mater.* **2023**, *33*, 2300425.

[14] W. Xu, W. Dong, J. Lin, K. Mu, Z. Song, J. Tan, R. Wang, Q. Liu, C. Zhu, J. Xu, L. Tian, *Advanced Science* **2024**, *n/a*, 2400466.

[15] B. Yuan, B. Zhao, Q. Wang, Y. Bai, Z. Cheng, Z. Cong, Y. Lu, F. Ji, F. Shen, P.-F. Wang, X. Han, *Energy Storage Mater.* **2022**, *47*, 288-296.

[16] X. Yang, K. Doyle-Davis, X. Gao, X. Sun, *eTransportation* **2022**, *11*, 100152.

[17] X. Da, J. Chen, Y. Qin, J. Zhao, X. Jia, Y. Zhao, X. Deng, Y. Li, N. Gao, Y. Su, Q. Rong, X. Kong, J. Xiong, X. Hu, S. Ding, G. Gao, *Adv. Energy Mater.* **2024**, *14*, 2303527.

[18] Y. Zhao, L. Li, D. Zhou, Y. Ma, Y. Zhang, H. Yang, S. Fan, H. Tong, S. Li, W. Qu, *Angew. Chem. Int. Ed.* **2024**, *63*, e202404728.
